# Inhibition of Shp2 ameliorates monocrotaline-induced pulmonary arterial hypertension in rats

**DOI:** 10.1186/s12890-018-0700-y

**Published:** 2018-08-07

**Authors:** Yusheng Cheng, Min Yu, Jian Xu, Mengyu He, Hong Wang, Hui Kong, Weiping Xie

**Affiliations:** 1 Department of Respiratory and Critical Care Medicine, Yijishan Hospital of Wannan Medical College, 2 Zeshan West Road, Wuhu, 241001 Anhui China; 2 Department of Respiratory and Critical Care Medicine, the First Affiliated Hospital of Nanjing Medical University, 300 Guangzhou Road, Nanjing, 210029 Jiangsu China

**Keywords:** Shp2, Pulmonary vascular remodeling, Pulmonary hypertension

## Abstract

**Background:**

Src homology 2 containing protein tyrosine phosphatase (PTP) 2 (Shp2) is a typical tyrosine phosphatase interacting with receptor tyrosine kinase to regulate multiple signaling pathways in diverse pathological processes. Here, we will investigate the effect of Shp2 inhibition on pulmonary arterial hypertension (PAH) in a rat model and its potential cellular and molecular mechanisms underlying.

**Methods:**

Monocrotaline (MCT)-induced PAH rat model was used in this study. Phps-1, a highly selective inhibitor for Shp2, was administered from 21 days to 35 days after MCT single-injection. Microcatheter method was applied to detected hemodynamic parameters. Histological methods were used to determine PVR changes in PAH rats. Moreover, cultured pulmonary artery smooth muscle cells (PASMCs) treated by platelet-derived growth factor (PDGF) with or without Phps-1 was used to investigate the potential cellular and molecular mechanisms underlying in vitro.

**Results:**

Inhibition of Shp2 significantly attenuated MCT-induced increases of mean pulmonary arterial pressure (mPAP), right ventricular systolic pressure (RVSP) and right ventricular hypertrophy (RVH) in rats. Shp2 inhibition effectively decreased thickening of pulmonary artery media and cardiomyocyte hypertrophy as well as perivascular and myocardial fibrosis in MCT-treated rats. Moreover, Shp2 inhibition ameliorated muscularization of pulmonary arterioles in MCT-induced PAH rats. Shp2 inhibition significantly reduced platelet-derived growth factor (PDGF)-triggered proliferation and migration of human pulmonary artery smooth muscle cells (PASMCs), which might be attributed to the inactivations of Akt and Stat3 pathways.

**Conclusions:**

Shp2 contributes to the development of PAH in rats, which might be a potential target for the treatment of PAH.

## Background

Pulmonary arterial hypertension (PAH) is a life-threatening disorder, characterized by progressive pulmonary vascular remodeling (PVR) leading to increased pulmonary vascular resistance, right heart failure and ultimately premature death [1]. Recent findings have suggested that abnormal vasoconstriction, vascular inflammation and remodeling, endothelial dysfunction and thrombotic arteriopathy are typical features of PAH [2]. By far, PVR, mainly caused by aberrant proliferation of pulmonary vascular cells and abnormal formation of extracellular matrix, have been recognized as the pathological features of PAH [3].

Although considerable advances have been made in treating PAH during the past decades, mortality in patients with PAH remains high [4]. One major reason is that recent available medications mainly tackle the pulmonary artery endothelial dysfunction and leave the vascular remodeling suboptimally inhibited [5]. Currently, pulmonary arterial smooth muscle cells (PASMCs) with apoptosis-resistant phenotype are well accepted as one major contributor for PVR changes in PAH [2]. Thus, PASMCs is recognized as a promising target for intervention of PAH [6]. Platelet-derived growth factor (PDGF) is an important growth factor for proliferation and migration of PASMCs through activating receptor tyrosine kinases, which increases significantly and promotes PVR in patients with PAH [3]. Several clinical trials targeting PDGF signaling have been carried out to treat patients with PAH. For example, Imatinib and PK10453, two novel PDGF receptor tyrosine kinase inhibitors, are found to improve PVR efficiently [7, 8]. In this context, receptor tyrosine kinases provide new strategies for improving PAH [9, 10].

Src homology 2 containing protein tyrosine phosphatase (PTP)-2 (Shp2) is a member of the non-receptor protein tyrosine phosphates family, which has drawn growing attentions in recent years for its interaction with receptor tyrosine kinase to regulate multiple signaling pathways linked with cellular development and diverse pathological processes [11]. Shp2 is widely expressed in different tissues and enhances the migration of aortic vascular smooth muscle cells (SMCs) isolated from adult rats [12, 13]. Moreover, Shp2 promotes the activation of receptor protein tyrosine kinase of PDGF receptor β (PDGFR β) signal pathway in SMCs [14]. Interestingly, Shp2 is found to be a target of miR-204 and sustains proliferation and anti-apoptotic feature of PASMCs in PAH [15]. However, there is no data to elucidate whether Shp2 contributes to PVR changes in PAH. In this study, we investigated the effects of Shp2 inhibition on PAH in rats and its potential cellular and molecular mechanisms underlying.

## Methods

### Experimental animals

This study was approved by the animal ethical and welfare committee of Nanjing Medical University (Approval No. 1601271). Male Sprague-Dawley (SD) rats (weight between 200 and 250 g, 5–7 weeks age) were purchased from Bikai experiment animals center (Shanghai, China). All the animals were housed in climate-controlled conditions with 12 h light and 12 h dark cycle and had free access to chow and water for 5 days. Monocrotaline (MCT)-induced PAH rat model was used in this study. Rats were subcutaneously injected with a single dose of MCT (40 mg/kg, Sigma, St, Louis, MO). The dosage of MCT used in this study was determined by our pre-experiment work to reduce high mortality of rats after MCT administration. Twenty one days after MCT administration, rats were then intraperitoneally injected with Phps-1 (1 mg/kg, Sigma, St, Louis, MO) (a highly selective inhibitor for Shp2**)** or a vehicle every other day. At the end of 35 days, all rats were examined.

### Hemodynamic analysis

Thirty-five days after a single injection of MCT, all rats were anaesthetised by an intramuscular injection of a cocktail of ketamine (90 mg/kg) and xylazine (10 mg/kg). The internal jugular vein was exposed by a 2–3 cm incision over the right ventral neck area. A polyethylene catheter connected to a pressure transducer was inserted into the right external jugular vein and threaded into the right ventricle, and then into right ventricular and pulmonary artery to measure systolic pressure (RVSP) and mean pulmonary artrial pressure (mPAP). Another catheter was inserted into left carotid artery to measure systemic arterial pressure (SAP) by a polygraph system (MP100, BIOPAC System, Inc., Santa Barbara, CA, USA). After homodynamic measurements, all anaesthetised rats were euthanized by exsanguination.

### Histological analysis

Right ventricle and distal lungs of rats removed en bloc were fixed in 4% paraformaldehyde, and then sectioned at 5-μm for subsequntly stainings. Hematoxylin and eosin staining was used to determine cardiomyocyte hypertrophy and pulmonary artery media thickness (PAWT). More than 20 images of distal pulmonary arterioles per rat (diameter between 30 and 100 μm) were captured using a microscopic digital camera and analysis program (Becton Dickinson). The PAWT is defined as the distance between inner and outer elastic lamina. Vessel external diameter (ED) was determined. The relative PAMT (%) was calculated as 100 × 2PAMT/ED. Cardiomyocyte hypertrophy was determined by cross sectional area (CSA) of cardiomyocyte. Moreover, sectioned lung tissues and myocardial tissues were stained with Masson’s trichrome staining which indicated the scales of collagen deposition, and the results were assessed by Image J software. Right ventricular hypertrophy (RVH) was presented as the ratio of right ventricle (RV) weight/ Left ventricle (LV) + septum (S) weight [16].

### Cell culture and reagents

PASMCs and cell culture medium components were obtained from ScienCell Research Laboratories (San Diego, CA), and used according to the manufacturer’s instructions. Briefly, PASMCs were maintained in smooth muscle cell medium (SMCM) supplemented with 2% fetal bovine serum (FBS), 1% penicillin / streptomycin and 1% mixed growth factors at 5% CO_2_ and 37 °C. The cells were starved for 24 h in SMCM with serum and growth factors free before subsequent experiments. Platelet derived growth factor (PDGF)-BB was obtained from Roche Group (Roche, USA).

### Immunofluorescent assay

Fresh lung tissues were embedded in OCT compound (Sakura Finetek, Torrance, CA, USA) and frozen in a dry-ice acetone bath. Seven-μm sectioned tissues were kept at − 80 °C until analysis. Tissue sections were incubated for 24 h at 4 °C with rabbit monoclonal anti-α-smooth muscle actin (α-SMA) antibody (1: 200, Abcam, USA) along with mouse monoclonal anti-CD31 antibody (2 μg/ml,Abcam, USA). After washes, sections were incubated with Alexa Fluor 488 goat anti-mouse IgG (Invitrogen, Molecular Probes, Carlsbad, CA, USA; 1:1000) and Alexa Fluor 594 goat anti-rabbit IgG (Invitrogen; 1:1000) at room temperature for 1 h. The immunolabeled frozen sections were detected with Pannoramic Viewer (3DHISTECH 1.15.3). At least 60–80 distal pulmonary arterioles per rat were assessed. The muscularization of distal pulmonary arterioles was determined by calculating the percent of arteries that were fully, partially and not muscularuized.

### Cell proliferation assay

Cell counting kit-8 method was used to determine PASMCs cell proliferation as described previously [17]. Briefly, PASMCs at 10,000 cells each well were planted into 96-well plates in 100 μl of culture medium and incubated with different treatment for 24 h. After treatment, 10 μl of CCK-8 reagent (Dojindo Molecular Technologies, Kumamoto, Japan) was added to each well 4 h before the end of incubation. The optical density value (OD) of each sample was measured at a wavelength of 450 nm on a microplate reader (Thermo Scientific, CA, USA). The results of cell viability measurement were expressed as the absorbance at OD 450.

### Transwell migration assay

Migration of PASMCs was determined by Transwell assay, as described previously [18]. Shortly, 50,000 cells were seeded on the top of each polycarbonate filter with 8-μm pores (Corning) in 0.1 ml of basal medium, and then stimulated by PDGF (20 ng/ml) with or without Phps-1 (20 μM) for 12 h. Following exposure, the cells were fixed in 4% paraformaldehyde for 30 min and stained with 5% crystal violet (Beytime, China) for 30 min. Unmigrated cells were then scraped off the top of the filter. For each filter, at least five randomly chosen fields were imaged to obtain a total cell count.

### Western blotting analyses

Isolated lungs tissues or PASMCs were lysed in RIPA Lysis Buffer (Pierce Inc.) supplemented with 1% protease inhibitor cocktail (Roche) and 1 mM phenylmethylsulfonyl fluoride (PMSF). Serum-starve PASMCs were stimulated by PDGF (20 ng/ml) for 15 min with or without Phps-1 (20 μM) pre-treatment for 30 min. And then, proteins were detected by western blotting method. Lysates were centrifuged at 12,000 rpm at 4 °C for 15 min, and supernatants were collected for subsequent western blot analysis. Protein samples were separated on a SDS-PAGE gel and polyvinylidene fluoride membranes. Next, the membranes were blocked with 5% non-fat milk powder in Tris-buffered saline containing 0.1% Tween 20 for 1 h at room temperature, and then, the membranes were probed overnight at 4 °C with primary antibody: phospho-Shp2 (Santa Cruz, CA, USA), Shp2 (Santa Cruz, CA, USA), Akt (Cell Signaling Technology, Danvers, USA), phospho-Akt (p-Akt) (Cell Signaling Technology, Danvers, USA), transforming growth factor-β (TGF-β) (Cell Signaling Technology, Danvers, USA), phospho-Stat3 (Cell Signaling Technology, Danvers, USA), Stat3 (Cell Signaling Technology, Danvers, USA). Then, the blot was incubated with the appropriate horseradish-peroxidase (HRP)-conjugated rabbit IgG (Santa Cruz, CA, USA) or HRP-conjugated goat IgG as a secondary antibody (Santa Cruz, CA, USA). The signals were visualized using an enhanced chemilu-minescence (ECL) reagent kit (Thermo fisher scientific, Waltham, USA), and analyzed with Bio-Rad Gel Doc/Chemi Doc Imaging System and Image J software..

### Statistics

All values are presented as means ± standard deviation (SD). Data were analyzed using GraphPad prism 6.0. Comparisons were made using one-way analysis of variance (ANOVA) with Bonferroni multiple comparisons test. Statistical significance was defined as *P* < 0.05.

## Results

### Shp2 inhibition improves mPAP, RVSP and RVH in MCT-induced PAH rats

**Fig. 1:**
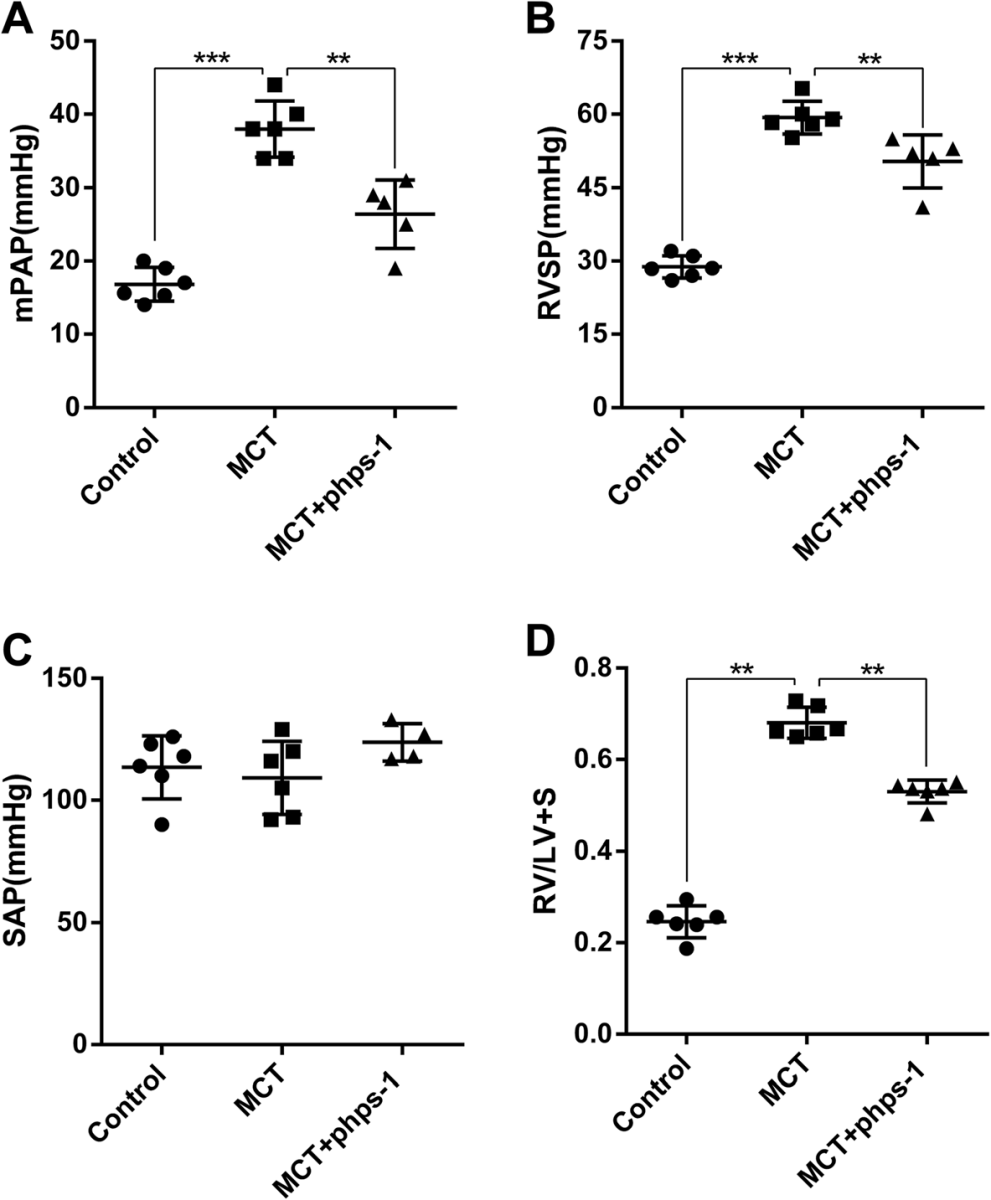
Shp2 inhibition improves mPAP, RVSP and RVH, without affecting SAP in MCT-induced PAH rats. **a** Phps-1 inhibited MCT- induced increases of mPAP and RVSP **b** (*n* = 6 for Control or MCT group, *n* = 5 for MCT + Phps-1group) without affecting SAP **c** (*n* = 6 for Control or MCT group, *n* = 4 for MCT + Phps-1 group). **d** Phps-1 suppressed MCT- induced increase of RVH, as indicated by RV/LV + S (*n* = 6 for each group). Data was presented as means ± standard deviation (SD), ** *P* < 0.01 and *** *P* < 0.001

In PAH rat, curatively treated with Shp2 inhibitor Phps-1markedly decreased MCT-induced elevation in mPAP (Fig. 1a) as well as RVSP (Fig. 1b). However, the Phps-1 had not significant effect on SAP (Fig. 1c). Similarly, Phps-1 partially reversed MCT induced increase of RVH, as indicated by weight ratio of RV/ (LV + S) (Fig. 1d). Thus, Shp2 inhibition effectively reduced mPAP, RVSP and RVH in MCT-induced PAH rats.

### Shp2 inhibition attenuates MCT-induced thickening of PAMT and perivascular fibrosis

**Fig. 2:**
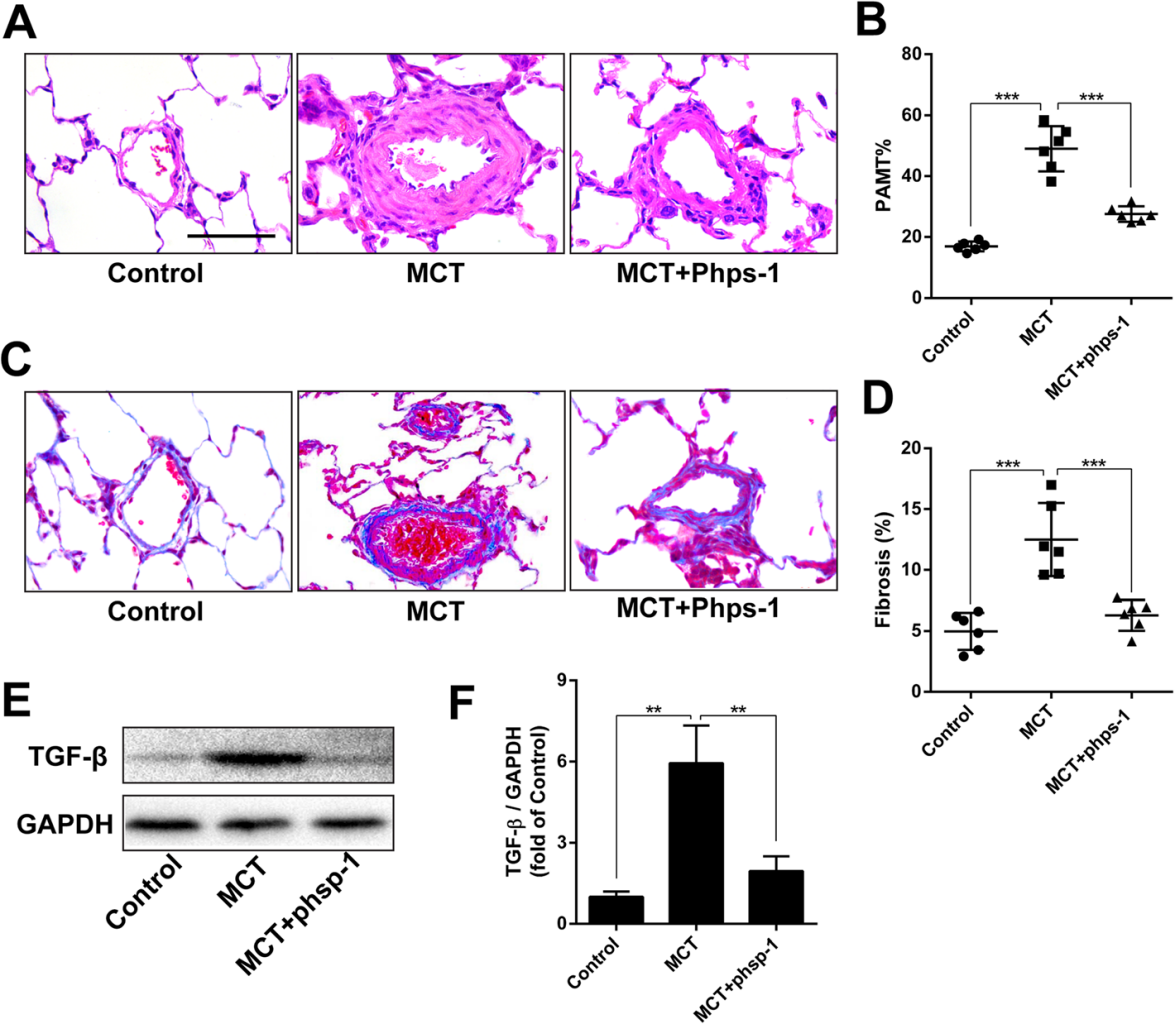
Shp2 inhibition attenuates MCT-induced thickening of PAMT and perivascular fibrosis. **a** Representative images of hematoxylin and eosin staining for PAMT, Scale bar = 30 μm. **b** Quantification of PMWT (*n* = 6 for each group). **c** Representative images of Masson’s trichrome staining for detecting perivascular fibrosis (blue), Scale bar = 30 μm. **d** Hemi-quantification of perivascular fibrosis (*n* = 6 for each group). **e** and **f** Phps-1 reversed MCT-induced overexpression of TGF-β in lungs (*n* = 3). Data was presented as means ± standard deviation (SD), ** *P* < 0.01 and *** *P* < 0.001

As shown in Fig. 2a and b, the PAMT was significantly thickened in MCT-induced PAH rats, which was alleviated by Phps-1 treatment. Masson’s trichrome staining showed that Phps-1 reversed MCT-induced perivascular fibrosis (Fig. 2c and d). Moreover, Phps-1 significantly inhibited MCT-induced overexpression of TGF-β in lung tissues (Fig. 2e and f). Therefore, inhibition of Shp2 significantly improved thickening of PAMT and perivascular fibrosis in MCT-induced PAH rats.

### Shp2 inhibition ameliorates muscularization of pulmonary arterioles in rats

**Fig. 3:**
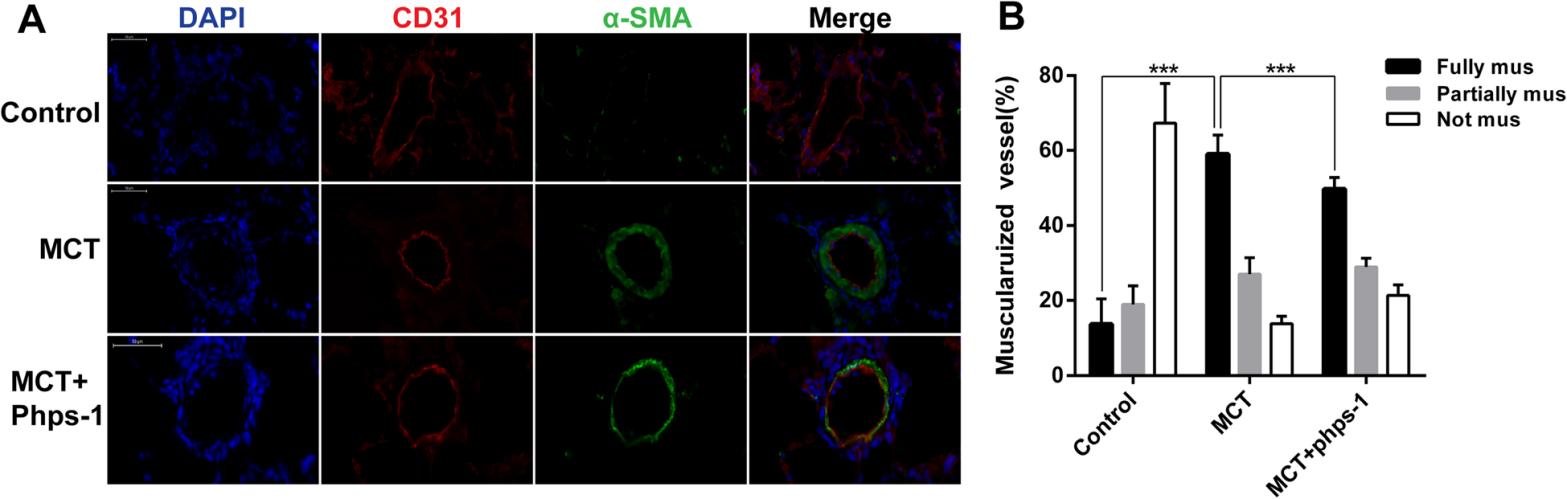
Shp2 inhibition ameliorates muscularization of pulmonary arterioles in MCT induced PAH rats. **a** Phps-1 decreased α-SMA expression of lungs in MCT induced PAH. Representative images of immunofluorescent stainings for CD31 (red) and α-SMA (green) in lungs were showed. Nucleus (blue) was stained with DAPI. Scale bar = 50 μm. **b** The muscularization of distal pulmonary arterioles was determined by calculating the percent of arteries that were fully (> 75%), partially (25–75%) and not muscularuized (< 25%) (*n* = 4–6). Data was presented as means ± standard deviation (SD), *** *P* < 0.001

The muscularization of pulmonary arterioles was evaluated by immunofluorence of smooth muscle cell marker α-SMA. As shown in Fig. 3a, α-SMA was over-expressed in PAH group compared to control group, which was remarkably inhibited by the treatment of Phps-1. Statistical analysis indicated that Phps-1 treatment significantly decreased portion of fully muscularized pulmonary arteries induced by MCT (Fig. 3b).

### Shp2 inhibition reduces cardiomyocyte hypertrophy and myocardial fibrosis in MCT- induced PAH rats

**Fig. 4:**
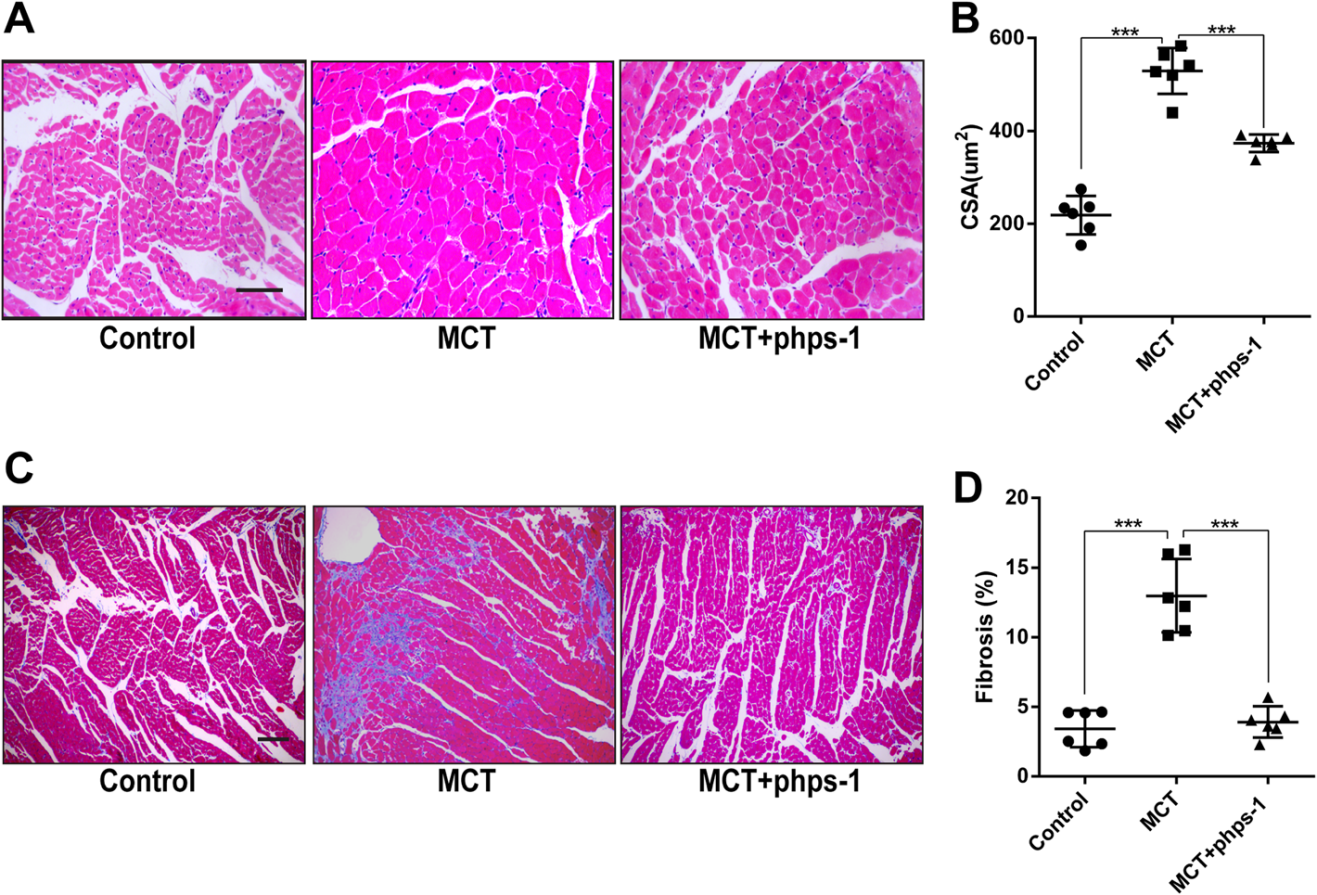
Shp2 inhibition reduces cardiomyocyte hypertrophy and normalized myocardial fibrosis in MCT-induced PAH. **a** Representative images of hematoxylin and eosin staining for cardiomyocyte hypertrophy. Scale bar = 50 μm. **b** Quantification of CSA (*n* = 6 for each group). **c** Representative images of Masson’s trichrome staining for detecting myocardial fibrosis (blue). Scale bar = 50 μm. **d** Quantification of myocardial fibrosis (*n* = 6 for each group). Data was presented as means ± standard deviation (SD), *** *P* < 0.001

Persistent elevated pulmonary arterial pressure could lead to cardiomyocyte hypertrophy and fibrosis in PAH. CSA of cardiomyocyte analysis based on hematoxylin and eosin staining showed that Phps-1 significantly suppressed MCT-induced cardiomyocyte hypertrophy (Fig. 4a and b). Morever, semiquantitative analysis of myocardial fibrosis by Masson’s trichrome staining demonstrated that Phps-1 reversed myocardial fibrosis in MCT-induced PAH rats (Fig. 4c and d).

### Shp2 inhibition decreases PDGF-induced proliferation and migration of PASMCs

**Fig. 5:**
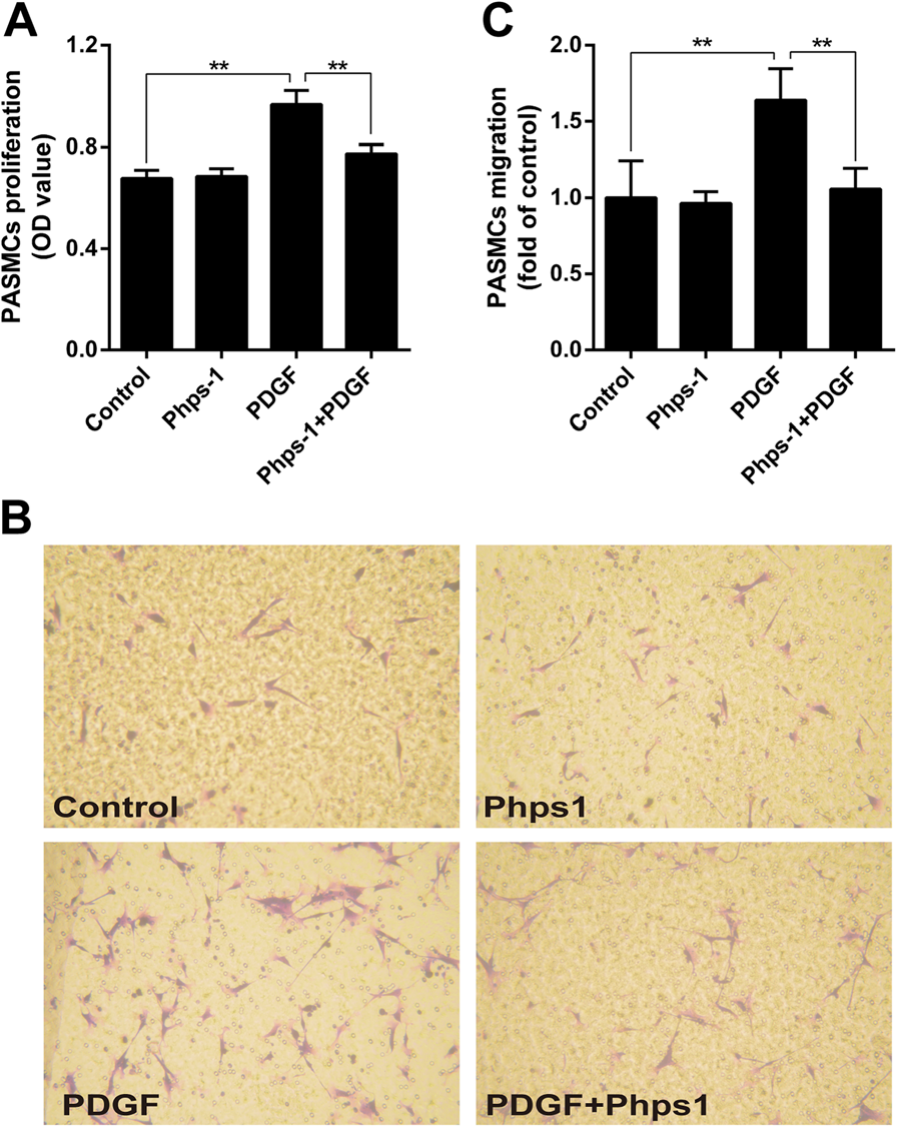
Shp2 inhibition decreases PDGF-induced proliferation and migration of human PASMCs. **a** Phps-1 (20 μM) inhibited PDGF (20 ng/ml)-induced proliferation of human PASMCs. CCK-8 assay was used (*n* = 5). Data was from five independent tests. **b** Representative images of PASMCs migration. Transwell migration method was used. **c** Phps-1 (20 μM) significantly inhibited PDGF (20 ng/ml)-induced migration of human PASMCs (*n* = 4). Data was presented as means ± standard deviation (SD), ** *P* < 0.01

PDGF is a key growth factor promoting the proliferation and migration of PASMCs in the development of PAH. In cultured human PASMCs, suppressing the activation of Shp2 by selective inhibitor Phps-1 blocked PDGF-stimulated cells proliferation (Fig. 5a). Additionally, transwell assay showed that Phps-1 efficiently inhibited PDGF-induced migration of PASMCs (Fig. 5b and c).

### Shp2 inhibition blocks PDGF-stimulated activations of Akt and Stat3 signaling

**Fig. 6:**
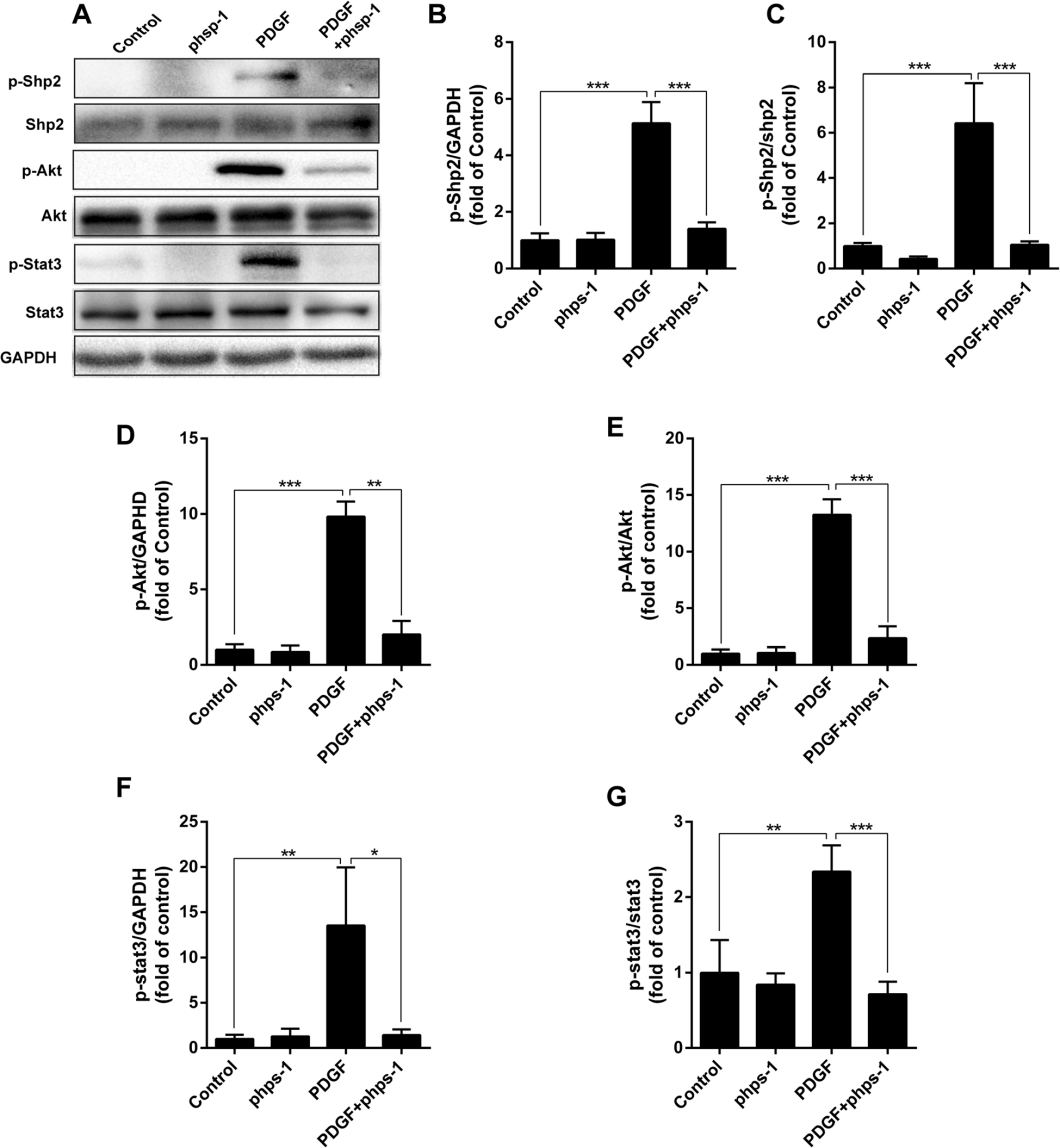
Inhibition of Shp2 inhibits PDGF-stimulated activations of Akt and Stat3 pathways. **a** Representative images of blotting for p-Shp2/Shp2, p-Akt/Akt and p-Stat3/Stat3 in PASMCs. Quantifications of p-Shp2 (**b**), the ratio of pShp2/Shp2 (**c**), p-Akt (**d**), the ratio of p-Akt /Akt (**e**), p-Stat3 (**f**) and the ratio of p-Stat3 /Stat3 (**g**) (*n* = 3). Data was presented as means ± standard deviation (SD), *n* = 3, *P < 0.05, ** *P* < 0.01 and *** *P* < 0.001

As shown in Fig. 6, PDGF dramatically activated Shp2 by enhancing the phosphorylation of Shp2. Akt and Stat3 are two important kinases involved in PDGF receptor signal transduction. As expected, PDGF also induced potent phosphorylation of Akt and Stat3 in cultured human PASMCs. However, these stimulant effects of PDGF on phosphorylation of Shp2, as well as Akt and Stat3 were effectively blocked by Phps-1 (Fig. 6).

## Discussion

The main findings of this study suggest that Shp2 is an important contributor to the development of PAH. Pharmacological inhibition of Shp2 by its highly selective inhibitor Phps-1 markedly decreased MCT-induced elevation of mPAP and RVSP, as well as MCT-induced PVR, including decreasing PAMT, suppressing perivascular collagen deposit and ameliorating muscularization of distal pulmonary arterioles. In addition, data from in vitro studies suggests that Akt and Stat3 pathways are involved in the beneficial effects of Shp2 inhibition on PDGF-induced proliferation and migration of human PASMCs.

Currently, several tyrosine kinase inhibitors for improving remodeling pulmonary vascular in PAH have been investigated [19]. For example, Imatinib, a potent receptor tyrosine kinase inhibitor for treatment of chronic myeloid leukemia, was found to effectively reverse PVR in animal models of PAH and improved the hemodynamics and exercise capacity in PAH patients [20, 21]. Sorafenib, a mutikinase inhibitor of tyrosine kinases as well as serine-threonine kinases, prevented PVR and improved cardiac functions in experimental model of pulmonary hypertension [22]. Therefore, receptor tyrosine kinases provide promising new targets for improving PAH. As a key mediator for several receptor tyrosine kinases and SRC-family kinases, Shp2 regulates signal transduction of diverse growth factors and hormones relating to fundamental cellular function [23–25]. Phps-1 is found to be a highly selective inhibitor for Shp2, which binds the catalytic site of Shp2 and blocks Shp2-dependent signaling [26]. For example, Phps-1 effectively inhibits TGF-β1-induced epithelial-mesenchymal transition in lung epithelial A549 cells [27]. Additionally, Phps-1 is reported to alleviate airway inflammation and airway hyper-responsiveness in allergic mice [28]. Of note, the beneficial effect of tyrosine kinase antagonist in PAH was accompanied by dose-dependent decreases in SAP [29]. As a typical tyrosine phosphatase, Shp2 interacts with tyrosine kinase to regulate proliferation and migration of smooth muscle cells [11]. Thus, theoretically, Shp2 inhibition might influence SAP in PAH. However, in our study, Phps-1 improved mPAP, RVSP and RVH in MCT-induced PAH rats with no significant effect on SAP. This may be attributed to limited dosage of Phps-1 used in the present study. Moreover, this result also suggests Phps-1 may have tissue-selective distribution in pulmonary circulation rather than systemic circulation. Improvement of PVR is one of major goals of current medications for PAH, which delays increases of pulmonary arterial pressure and right heart afterload and decreases mortality of PAH ultimately [5]. In the present study, inhibition of Shp2 effectively reduced increases of PAMT and perivascular fibrosis in PAH rat lungs. Moreover, Shp2 inhibition ameliorated pulmonary arterioles muscularization in rats. Therefore, inhibition of Shp2 effectively inhibited PVR in MCT-induced PAH rats.

Myocardial hypertrophy and fibrosis caused by pressure-overload of RV results in dysfunction of RV in PAH. [30]. In this study, we revealed that inhibition of Shp2 suppressed myocardial hypertrophy and reversed myocardial fibrosis in MCT-induced PAH rats. So far, dysfunction of Shp2 has been demonstrated to cause disorders of myocardium structure and function. Loss-of-function mutations in PTPN11 gene, which encodes the Shp2, leads to congenital heart disease and adult-onset heart hypertrophy [31]. Hypertrophic cardiomyopathy caused by Shp2 dysfunction is mediated by aberrant activation of Akt, focal adhesion kinase and mammalian target of rapamycin. Inhibition of Shp2 remarkably prevents heart hypertrophy [32]. In summary, Shp2 contributes to maladaptive remodeling of RV in MCT-induced PAH rats.

PDGF is an important growth factor in the development of PAH, stimulating proliferation of PASMCs by activating Stat3 and Akt signal pathways [33, 34]. In the present study, inhibition of Shp2 significantly inhibited PDGF-stimulated proliferation and migration of human PASMCs. It is reported that Shp2 is necessary for the activation of Akt pathway in vascular smooth muscle cell hypertrophy [35]. Meanwhile, Shp2 promotes PDGF-stimulated Akt activation in fibroblasts [23]. In this study, we found inhibition of Shp2 effectively blocked PDGF-induced activations of Akt and Stat3 in PASMCs. Thus, Akt and Stat3 pathways are involved in the beneficial effects of Shp2 inhibition on PDGF-induced proliferation and migration of human PASMCs.

However, there are several limitations in this study. First, it did not clarify whether inhibition of Shp2 could modulate other growth factors including basic fibroblast growth factor (FGF), insulin-like growth factor-1 (IGF-1) and epidermal growth factor (EGF) in spite of Shp2 inhibition significantly decreased TGF-β expression in MCT-induced PAH rat lungs. Those growth factors are reported to associate with the development of PVR [36–39]. Second, the effect of Shp2 on fibroblast cells is not investigated. However, Shp2 inactivation markedly decreased peri-vascular cells proliferations in lungs of PAH (data not shown). Finally, the mechanisms of Shp2 regulating PVR remain to be further fully investigated.

## Conclusions

Our findings demonstrated that inhibition of Shp2 ameliorates MCT-induced PAH in rats, which might be a potential target for the treatment of PAH.

## Abbreviations

CSA: Cross-sectional area
ED: External diameter
EGF: Epidermal growth factor
FBS: Fetal bovine serum
FGF: Fibroblast growth factor
IGF-1: Insulin-like growth factor-1
mPAP: Mean pulmonary artrial pressure
OD: Optical density
PAH: Pulmonary arterial hypertension
PASMCs: Pulmonary arterial smooth muscle cells
PAWT: Pulmonary artery media thickness
PDGF: Platelet-derived growth factor
PDGFR β: PDGF receptor β
PMSF: Phenylmethylsulfonyl fluoride
PVR: Pulmonary vascular remodeling
RVH: Right ventricular hypertrophy
RVSP: Right ventricular systolic pressure
SAP: Systemic arterial pressure
Shp2: Src homology 2 containing protein tyrosine phosphatase (PTP)-2
SMCM: Smooth muscle cell medium
TGF-β: Transforming growth factor-β
α-SMA: α-smooth muscle actin
